# Technical Factors Influencing Cone Packing Density Estimates in Adaptive Optics Flood Illuminated Retinal Images

**DOI:** 10.1371/journal.pone.0107402

**Published:** 2014-09-09

**Authors:** Marco Lombardo, Sebastiano Serrao, Giuseppe Lombardo

**Affiliations:** 1 Fondazione G.B. Bietti IRCCS, Rome, Italy; 2 CNR-IPCF Unit of Support Cosenza, Rende, Italy; 3 Vision Engineering Italy srl, Rome, Italy; University of Modena and Reggio Emilia, Italy

## Abstract

**Purpose:**

To investigate the influence of various technical factors on the variation of cone packing density estimates in adaptive optics flood illuminated retinal images.

**Methods:**

Adaptive optics images of the photoreceptor mosaic were obtained in fifteen healthy subjects. The cone density and Voronoi diagrams were assessed in sampling windows of 320×320 µm, 160×160 µm and 64×64 µm at 1.5 degree temporal and superior eccentricity from the preferred locus of fixation (PRL). The technical factors that have been analyzed included the sampling window size, the corrected retinal magnification factor (RMF_corr_), the conversion from radial to linear distance from the PRL, the displacement between the PRL and foveal center and the manual checking of cone identification algorithm. Bland-Altman analysis was used to assess the agreement between cone density estimated within the different sampling window conditions.

**Results:**

The cone density declined with decreasing sampling area and data between areas of different size showed low agreement. A high agreement was found between sampling areas of the same size when comparing density calculated with or without using individual RMF_corr_. The agreement between cone density measured at radial and linear distances from the PRL and between data referred to the PRL or the foveal center was moderate. The percentage of Voronoi tiles with hexagonal packing arrangement was comparable between sampling areas of different size. The *boundary* effect, presence of any retinal vessels, and the manual selection of cones missed by the automated identification algorithm were identified as the factors influencing variation of cone packing arrangements in Voronoi diagrams.

**Conclusions:**

The sampling window size is the main technical factor that influences variation of cone density. Clear identification of each cone in the image and the use of a large buffer zone are necessary to minimize factors influencing variation of Voronoi diagrams of the cone mosaic.

## Introduction

Data on the density and packing arrangement of photoreceptors and their normal variance are relevant for evaluating the health of the photoreceptor mosaic and contribute to detecting and characterizing the degradation of mosaic quality due to pathologic alterations occurring in retinal diseases. Several methods have been reported for identification of cones in adaptive optics (AO) retinal images [1]–[13]. The precision of each new algorithm was in general assessed by comparing the cone locations with those determined manually or by an algorithm with known accuracy [12]–[16]. In most cases, careful manual analysis was shown to produce quite accurate cone identification. A moderate to high inter-individual variability (defined as the ratio of the standard deviation to the mean) of cone density, ranging between 12% and 20% between 250 and 1300 µm from the fovea, has been reported in studies of healthy populations [3]–[7], [10], [17]. Discrepancies between studies have been related both to demographic and technical factors, such as 1) the inclusion of subjects with different ages or eyes with different axial lengths and refractive corrections, 2) the instrument used for biometry, 3) the model eye used to estimate the retinal image size, 4) the use of individual correction of retinal magnification, 5) the use of foveal center or preferred locus of fixation (PRL) as reference point to define retinal eccentricities and 6) the sampling window area used to count cones. As regards demographic factors, it is well established that A) eyes with longer axial length have lower cone density compared with emmetropic eyes, B) cone density declines with increasing retinal eccentricity, C) density variation is higher close to the fovea than at increasing distance and D) the density of cones along the horizontal meridian is 10% higher than along the vertical meridian at each eccentricity [3]–[7], [10], [11], [13], [14], [17]. It is also accepted that there is a tendency towards decreasing density with increasing age [3], [14], [18].

The majority of studies evaluating the cone mosaic have been carried out using 50×50 µm to 64×64 µm sampling areas to calculate cone density. This approach was chosen in order to compare data acquired in patients with those shown by Curcio et al. [19]–[20] in cadaver eyes. On the other hand, recent papers have shown the reliability of using multiple complementary descriptors of the cone mosaic in sampling areas wider than currently used [13], [21], [22]. This approach has been shown to provide a more comprehensive view of the photoreceptor mosaic geometry. In previous work [15], we evaluated the effect of window size and orientation on cone density at four retinal parafoveal locations, showing that density estimates decrease with decreasing window size. The packing arrangement of cones, estimated using Voronoi diagrams, was not influenced by the sampling window size or its geometry.

The scope of this work was to investigate the influence of technical factors on the variation of cone density and Voronoi diagrams estimated in AO-flood illuminated images of the parafoveal cone mosaic in a population of healthy adults. These technical factors included the sampling window size, the corrected retinal magnification factor (RMF_corr_), the conversion from radial to linear distance from the PRL, the displacement between the PRL and foveal center and the manual checking of cone identification algorithm performance.

## Methods

All research procedures described in this work adhered to the tenets of the Declaration of Helsinki. The protocol was approved by the local ethical committee (Azienda Sanitaria Locale Roma A, Rome, Italy) and all subjects recruited gave written informed consent after a full explanation of the procedure. Inclusion criteria were an age >18 years old, no history of systemic or ocular diseases and no previous eye surgery. Subjects recruited for the study received a complete eye examination, including non-contact ocular biometry using the *IOL Master* (Carl Zeiss Meditec Inc, Jena, Germany) and retinal imaging using a *Spectralis* SLO/SD-OCT (Heidelberg Engineering GmbH, Heidelberg, Germany).

A flood-illuminated AO retinal camera (*rtx1*, Imagine Eyes, France) was used to acquire images of the cone mosaic. The imaging sessions were conducted after dilating the pupil with one drop of 1% tropicamide. In this study, image sequences of 40 frames each, subtending 4 degrees of visual angle, were recorded at 13 retinal locations, extending between 2 degree nasal, 2.5 degree temporal and 2.5° superior and inferior from the PRL in the right eye of each subject. During imaging, fixation was maintained by instructing the patient to fixate on the internal target of the instrument moved by the investigator.

A proprietary program provided by the manufacturer [23] has been used to correct for distortions within frames of the raw image sequence and to register and frame-average in order to produce a final image with enhanced signal-to-noise ratio. The final AO images acquired at 1.50 degree temporal and superior from the PRL (identified as the point with coordinates x = 0° and y = 0° and here used as the foveal reference point), have been used for subsequent analysis.

### Image analysis

We used the nonlinear formula of Drasdo and Fowler and the Gullstrand schematic model eye parameterized by the biometry measurements (corneal central curvature, anterior chamber central depth, axial length) to convert each final image from degrees of visual angle to micrometers on the retina [7], [13], [15], [24], [25]. The corrected magnification factor (RMF_corr_) was calculated for each eye in order to correct for the differences in optical magnification and thus retinal image size between eyes [13], [15], [18].

Three areas of different size (320×320 µm, 160×160 µm and 64×64 µm) were cropped from each final image and used for subsequent analysis of cone density and preferred packing arrangements of cones. The cone image labelling process was performed using an algorithm implemented with the Image Processing toolbox in Matlab (The Mathworks Inc, Natick MA, USA), as previously described in detail [1], [13], [15]. Cones were identified independently in each sampling window. The performance of the cone identification algorithm was verified by two expert investigators (ML and GL). When they fully agreed, the position of each cone, that was not automatically labeled, was digitized manually by one investigator by clicking on the cone and marking its location on the image. This procedure ensured that no cone was excluded. Cones whose edges were even partially outside the image section were not labeled. The *x*, *y* coordinates of the cones in each sampling window were then stored in a text array and used to calculate the cone density and packing arrangement. Cone counts were converted into local densities by calculating their number per square millimeter (cones/mm^2^).

Cone density was estimated at both radial and linear distance from the PRL along the horizontal and vertical meridians, which included 1.5 degree and 418 µm temporal and superior from the fovea respectively. The linear distance represented the average distance from the PRL converted from 1.5 degree in our study population. For each image, all the sampling windows were re-centered to this linear distance from the PRL. Density values obtained at 1.5 degree eccentricity were also calculated both with and without applying the RMF_corr_. In the latter case, a single RMF value was used for each sampling area in all subjects. The distance from the PRL to the foveal center has been further calculated. Although the foveal cones were not resolved by the *rtx1* instrument, registration of the montage image of the cone mosaic with the SLO/SD-OCT images helped to identify the foveal center (identified as the foveal pit) in each eye.

The cone packing arrangement was analyzed using Voronoi diagrams, as previously described [13], [15]. The Voronoi tessellation was implemented by the Matlab voronoi function using the two-dimensional coordinates of the labelled cones. Each Voronoi cell was colour-coded according to the number of neighbouring cones: gray = tetragonal (4*n*) arrangement, yellow = pentagonal (5*n*) arrangement, green = hexagonal (6*n*) arrangement; blue = heptagonal (7*n*) arrangement and white = octagonal (8*n*) arrangement. The Voronoi regions containing pixels that extended beyond the bounds of each section were excluded from further analysis, thus creating a thin buffer zone to minimize the *boundary effect* [26].

### Statistics

Retinal data were expressed as mean ± standard deviation. Statistics were performed using the SPSS software (SPSS Inc., version 17.0). The normal data distribution was verified using the P-P plot within the software. The analysis of variance and the Tukey pairwise test were used to test significance between the cone density measurements and the preferred packing arrangements of cones taken within windows of different size at the same retinal location. The coefficient of variation was used to analyze the variation of cone density between sampling windows of different size at each retinal location.

The intraclass correlation coefficient (ICC; two-way random effects model) was calculated in order to estimate the association between cone density values calculated within the various sampling windows. Bland-Altman analysis [27], [28] was used to assess the 95% limits of agreement (LoA) between the cone density values estimated between the various sampling window conditions along the same meridian.

A multiple regression analysis was used to determine the relationships between the eye biometry variables (AxL, SEr) and RMF_corr_. The statistical significance was set at *P*<0.05 for all the tests performed.

## Results

Fifteen adult subjects (6 males and 9 females) were recruited. The subjects were 21 to 46 years old (29.3±7.6 years), the spherical equivalent refraction (SEr) ranged between emmetropia and −6.25 D (mean, −2.87±2.21 D). The average axial length (AxL) was 24.56±1.36 mm (range, 21.66 to 27.04 mm). Normal eye examination was recorded in all cases.

The accuracy of the cone identification algorithm decreased as the sampling window size decreased. The mean percentage of manually identified cones was 0.1±0.5%, 1.3±0.7% and 4.9±3.8% for the 320×320 µm, 160×160 µm and 64×64 µm windows respectively.

### Influence of corrected magnification factor

The average RMF_corr_ was 0.282±0.015 mm/deg (range, 0.258–0.313 mm/deg). The absolute difference in cone density between the sampling windows of the same size with or without the use of individual RMF_corr_ was lower than 120 cones/mm^2^ (range, 75–117 cones/mm^2^; P>0.05) at both retinal locations.

### Influence of window size

The average cone density ranged between 30801±1313 cones/mm^2^ and 29023±2497 cones/mm^2^ across the various sampling windows at 1.5 degree temporal location. It ranged between 28231±2602 cones/mm^2^ and 26128±3185 cones/mm^2^ at 1.5 degree superior location. The measured cone density decreased with decreasing window size. The absolute difference between cone density estimated in 320×320 µm and 160×160 µm areas were lower than 450 cones/mm^2^ at both retinal locations (P = 0.09). The differences were greater (P = 0.07; ≤2100 cones/mm^2^) between density values calculated in 320×320 µm and 64×64 µm sampling areas. Table 1 summarizes cone density values and their 95% confidence levels at both retinal locations respectively. Cone density was on average 9% higher along the temporal than the superior location. The coefficient of variation increased from 4.2% to 8.6% and from 9.2% to 12.6% with decreasing window size from 320×320 µm to 64×64 µm at the temporal and superior locations respectively.

**Table 1:** Cone density values calculated in each sampling area at 1.5 degree temporal and superior eccentricity.

Sampling Area_Location	320×320_Temporal	160×160_Temporal	64×64_Temporal
*Mean*	30801	30374	29023
*St. Dev.*	1313	1807	2497
*Coefficient of Variation*	4.2%	5.9%	8.6%
*Lower 95% Confidence Level*	30463	29907	28378
*Upper 95% Confidence Level*	31140	30840	29668
**Sampling Area_Location**	**320×320_Superior**	**160×160_Superior**	**64×64_Superior**
*Mean*	28231	27929	26128
*St. Dev.*	2602	2969	3285
*Coefficient of Variation*	9.2%	10.6%	12.6%
*Lower 95% Confidence Level*	27559	27163	25280
*Upper 95% Confidence Level*	28902	28696	26976

### Influence of retinal conversion distance

The average linear distance from the PRL, corresponding to 1.5 degree eccentricity, was 418±19 µm, ranging from 387 to 454 µm. The displacement from 418 µm was statistically significantly correlated to SEr (r = −0.55; P = 0.03) and AxL (r = 0.68; P<0.01): the more myopic and, accordingly, the longer the eye, the higher the RMF_corr_ value. The absolute difference between cone density estimated at radial (1.5 deg) and linear (418 µm) distances from the fovea ranged between 98 and 362 cones/mm^2^. There were no statistically significant differences (P = 0.09) between cone density values calculated within windows of the same size along the same retinal meridian.

The average linear distance from the PRL to the foveal center was 27±15 µm, ranging from 11 to 46 µm. The displacement of the PRL from the foveal center was not correlated to SEr (r = 0.18; P = 0.58) or AxL (r = 0.19; P = 0.58).

### Correlation and agreement between cone density values

The ICC values between cone density calculated within sampling areas of different size ranged from 0.55 to 0.99 (P<0.001) and between 0.91 and 0.99 (P<0.001) along the temporal and superior locations respectively (tables 2 and 3). The lowest correlation values were found between cone density calculated in 320×320 µm and 64×64 µm windows at the temporal location (ICC<0.70).

**Table 2:** Correlation matrix of cone density values estimated within sampling windows of different size at the temporal location.

*	320/Deg/RMF_corr_	320/Deg/RMF_nocorr_	320/Um/RMF_corr_	160/Deg/RMF_corr_	160/Deg/RMF_nocorr_	160/Um/RMF_corr_	64/Deg/RMF_corr_	64/Deg/RMF_nocorr_	64/Um/RMF_corr_
**320/Deg/RMF_corr_**	1.00								
**320/Deg/RMF_nocorr_**	.99	1.00							
**320/Um/RMF_corr_**	.79	.80	1.00						
**160/Deg/RMF_corr_**	.70	.71	.92	1.00					
**160/Deg/RMF_nocorr_**	.70	.72	.92	.99	1.00				
**160/Um/RMF_corr_**	.68	.68	.93	.89	.88	1.00			
**64/Deg/RMF_corr_**	.58	.61	.77	.90	.90	.78	1.00		
**64/Deg/RMF_nocorr_**	.60	.63	.78	.90	.90	.78	.99	1.00	
**64/Um/RMF_corr_**	.55	.57	.88	.84	.84	.91	.81	.79	1.00

*Legend: window size/radial or linear distance/individual RMF_corr_ or not.

**Table 3:** Correlation matrix of cone density values estimated within sampling windows of different size at the superior location.

*	320/Deg/RMF_corr_	320/Deg/RMF_nocorr_	320/Um/RMF_corr_	160/Deg/RMF_corr_	160/Deg/RMF_nocorr_	160/Um/RMF_corr_	64/Deg/RMF_corr_	64/Deg/RMF_nocorr_	64/Um/RMF_corr_
**320/Deg/RMF_corr_**	1.00								
**320/Deg/RMF_nocorr_**	.99	1.00							
**320/Um/RMF_corr_**	.97	.97	1.00						
**160/Deg/RMF_corr_**	.99	.98	.95	1.00					
**160/Deg/RMF_nocorr_**	.99	.99	.95	.99	1.00				
**160/Um/RMF_corr_**	.99	.99	.99	.98	.98	1.00			
**64/Deg/RMF_corr_**	.96	.95	.92	.95	.95	.95	1.00		
**64/Deg/RMF_nocorr_**	.96	.95	.91	.95	.95	.95	.99	1.00	
**64/Um/RMF_corr_**	.95	.95	.98	.94	.94	.96	.93	.92	1.00

*Legend: window size/radial or linear distance/individual RMF_corr_ or not.

A high agreement was found between cone density calculated within the sampling windows of same size with or without using the RMF_corr_. The agreement between values estimated at radial and linear distance was moderate and the agreement calculated between sampling areas of different size was low. The same results were obtained across the temporal and superior retinal locations. The 95% limits of agreement for the temporal and superior locations are summarized in tables 4 and 5 respectively.

**Table 4:** Summary of Bland-Altmann analysis with 95% limits of Agreement (LoA) between the various sampling window conditions at the temporal location.

Effect of RMF_corr_	320 areas-radial dist.-RMF_corr_ vs RMF_nocorr_	160 areas-radial dist.-RMF_corr_ vs RMF_nocorr_	64 areas-radial dist.-RMF_corr_ vs RMF_nocorr_
*Mean*	30757	30334	28965
*Mean difference*	89	80	117
*Low 95% LoA*	−156	−205	−473
*High 95% LoA*	333	364	706
**Effect of conversion distance**	**320 areas-radial vs linear distance**	**160 areas-radial vs linear distance**	**64 areas-radial vs linear distance**
*Mean*	30621	30436	28934
*Mean difference*	362	−124	178
*Low 95% LoA*	−1570	−2111	−3099
*High 95% LoA*	2293	1863	3455
**Effect of size**	**320 vs 160 areas-radial distance**	**160 vs 64 areas-radial distance**	**320 vs 64 areas-radial distance**
*Mean*	30588	29698	29912
*Mean difference*	428	1351	1779
*Low 95% LoA*	−2087	−945	−2210
*High 95% LoA*	2942	3647	5767

**Table 5:** Summary of Bland-Altmann analysis with 95% limits of agreement (LoA) between the various sampling window conditions at the superior location.

Effect of RMF_corr_	320 areas-radial dist.-RMF_corr_ vs RMF_nocorr_	160 areas-radial dist.-RMF_corr_ vs RMF_nocorr_	64 areas-radial dist.-RMF_corr_ vs RMF_nocorr_
*Mean*	28191	27892	26077
*Mean difference*	79	75	103
*Low 95% LoA*	−150	−175	−420
*High 95% LoA*	308	325	625
**Effect of conversion distance**	**320 areas-radial vs linear distance**	**160 areas-radial vs linear distance**	**64 areas-radial vs linear distance**
*Mean*	28192	28062	26055
*Mean difference*	98	−265	146
*Low 95% LoA*	−1249	−1720	−2423
*High 95% LoA*	1444	1190	2714
**Effect of size**	**320 vs 160 areas-radial distance**	**160 vs 64 areas-radial distance**	**320 vs 64 areas-radial distance**
*Mean*	28080	27029	27179
*Mean difference*	302	1801	2013
*Low 95% LoA*	−853	−154	−51
*High 95% LoA*	1456	3756	4256

### Regression Model

**Figure 1:**
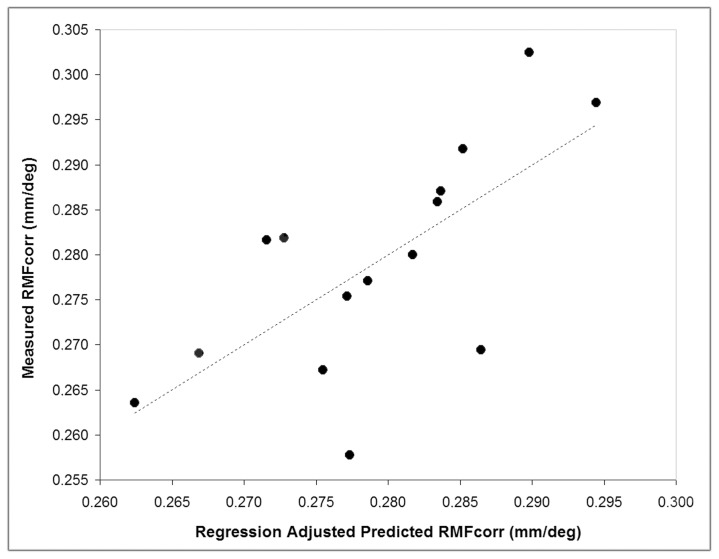
Correlation between the ocular biometry variables and the individual corrected retinal magnification factor (RMF_corr_). A 2-predictor model incorporating axial length and spherical equivalent refraction was developed in order to understand the correlation of biometry variables with the RMF_corr_. The model explains 70% of the variance of RMF_corr_ across the population (r = 0.70; P = 0.02).

The multiple linear regression model, including AxL and SEr as predictors, explained 70% of the variance of RMF_corr_ across the population (r = 0.70; P = 0.02), as shown in figure 1. The cone density differences between RMF_corr_ and RMF_predicted_ areas of 320×320 µm and 160×160 µm size were lower than 65- and 40-cones/mm^2^ at both retinal locations respectively. There were no differences (0 cones/mm^2^) when comparing data calculated within 64×64 µm areas.

### Voronoi analysis of the cone mosaic

**Figure 2:**
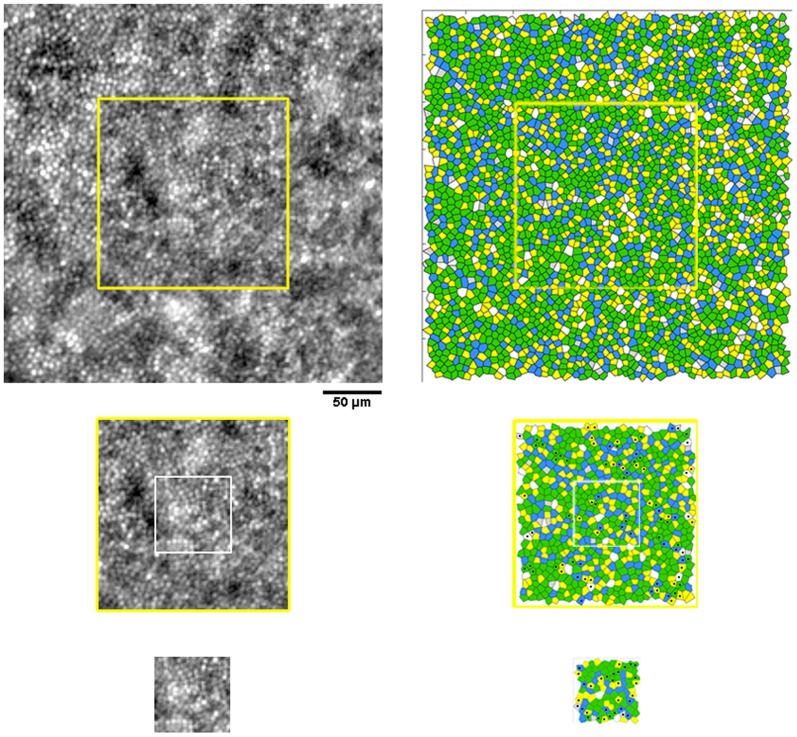
Images of the cone mosaic and corresponding Voronoi maps at 1.50 degree temporal eccentricity. Photoreceptor mosaic images acquired at 1.50 degree temporal eccentricity and corresponding Voronoi maps estimated within the three sampling areas of different size in case W10_S14. Scale bar is 50 µm. In this case, the differences in the % of hexagonal arrangement between sampling windows of different size were ≤1.7%. The black dots highlight the same Voronoi tiles that change their relative arrangement across sampling areas of different size.

**Figure 3:**
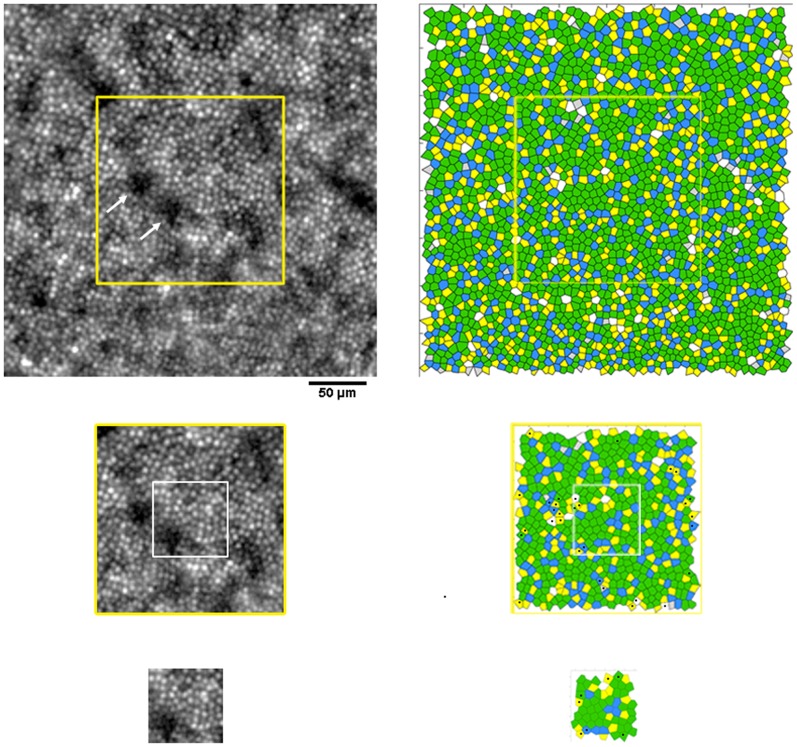
Images of the cone mosaic and corresponding Voronoi maps at 1.50 degree superior eccentricity. Photoreceptor mosaic images acquired at 1.50 degree superior location and corresponding Voronoi diagrams obtained from cone coordinates estimated within the three sampling areas of different size in case W10_S11. Scale bar is 50 µm. In this case, the difference in the % of hexagonal arrangement between sampling windows of different size were ≤6.5%. The black dots highlight the same Voronoi tiles that change their relative arrangement across sampling areas of different size. Presence of dark areas in the image of the cone mosaic (white arrows), the boundary effect and the manual selection of cones missed by automated counting influence the accuracy of Voronoi diagrams.

The percentage of hexagonal Voronoi tiles ranged between 44.5±3.5% and 47.5±5.3% at the temporal locations (P = 0.25) and between 49.1±7.9% and 50.7±4.6% at the superior locations (P = 0.95). Overall, as the sampling window decreased in size, the standard variation increased. At corresponding retinal locations, the maximum difference (3.1%; P = 0.25) was found between the 5*n* arrangements calculated between the 320×320 µm and 64×64 µm sampling areas at 418 µm temporal from the PRL. The differences between all the other non-hexagonal arrangements were ≤2% (P>0.05). A summary of the preferred cone packing arrangement calculated at radial and linear distances from the fovea is shown in tables 6 and 7 respectively. Overall, the variation of packing arrangements of cones was influenced by the *undersampling* effect (i.e., presence of any darks areas across the mosaic), the *boundary* effect and the manual selection of cones missed by the identification algorithm. Figures 2 and 3 show, in two representative cases, the Voronoi maps created within sampling windows of different size at the temporal and superior retinal locations respectively.

**Table 6:** Preferred packing arrangement of cones (average ± SD, %) calculated using Voronoi tiles at 1.5 degree temporal and superior eccentricities from the PRL.

*Preferred arrangement*	Temporal sampling windows (size)			Superior sampling windows (size)		
	320×320 µm	160×160 µm	64×64 µm	320×320 µm	160×160 µm	64×64 µm
*4n*	2.8±0.8	2.9±1.1	3.2±1.6	2.1±0.9	1.9±0.8	2.6±1.7
*5n*	26.9±0.5	27.3±1.5	26.4±3.2	24.7±1.3	25.4±1.9	26.9±3.8
*6n*	45.5±2.0	45.1±3.7	47.5±5.3	50.0±3.6	49.9±4.4	49.1±8.3
*7n*	20.5±0.8	20.5±1.4	19.5±3.7	20.1±0.9	20.1±1.7	19.7±4.2
*8n*	4.3±0.6	4.1±1.0	2.9±1.7	3.0±1.0	2.6±0.8	2.2±1.6

**Table 7:** Preferred packing arrangement of cones (average ± SD, %) calculated using Voronoi tiles at 418 µm temporal and superior eccentricities from the PRL.

*Preferred arrangement*	Temporal sampling windows (size)			Superior sampling windows (size)		
	320×320 µm	160×160 µm	64×64 µm	320×320 µm	160×160 µm	64×64 µm
*4n*	2.8±0.6	2.7±1.1	2.7±2.2	2.1±0.8	2.0±0.9	2.5±1.9
*5n*	26.6±0.9	27.6±1.6	29.8±4.6	24.6±1.4	24.8±1.9	26.9±5.0
*6n*	45.7±2.3	45.5±3.4	44.5±3.5	49.9±3.9	50.7±4.6	49.1±7.9
*7n*	20.8±0.7	20.6±1.5	20.3±3.0	20.3±0.9	20.0±1.3	19.4±3.6
*8n*	4.0±0.7	3.9±1.0	2.7±1.7	2.9±0.9	2.4±1.0	2.0±1.2

## Discussion

The clear identification of cones in a selected area of an AO image of the photoreceptor mosaic represents the key factor to reliably analyze the lattice quality. In addition, it is crucial to develop reliable descriptors to classify the normal distribution and spatial arrangement of the cones. A step that follows is to understand the source(s) of cone density variability in healthy populations of adults; in other words, to ascertain whether cone density variation can be ascribed to demographic or technical/methodological procedures and which of the above variables can have a greater influence. Knowledge of these factors would permit more accurate evaluation of pathological changes of the cone mosaic. The scope of our work was to understand the weighted influence of various technical factors on packing density estimates of parafoveal cones in AO flood illuminated retinal images of healthy subjects using a commercial device.

Cone density decreased with decreasing window size, as previously shown both in AO-flood illuminated and AO-SLO retinal images of the cone mosaic [15], [21]. Density values calculated within 64×64 µm areas showed 5–6% and 7–8% lower values than within 160×160 µm and 320×320 µm windows at 1.5 degree temporal and superior locations. The absolute differences between the largest and smallest sampling windows were lower than 2100 cones/mm^2^, approaching statistical significance. The 95% confidence level of cone density calculated in 64×64 µm areas did not overlap values obtained across 320×320 µm windows. The intersubject variation in density increased as the window size decreased from 320×320 µm to 64×64 µm and density values were 9% lower at the superior than at the temporal retinal location. Cone density estimated in areas of different size showed moderate to low agreement; it is therefore not recommended to compare density between different sampling areas in clinical studies. The error increases when comparing density calculated across 64×64 µm (or smaller) sampling areas with values calculated in windows much larger than 64×64 µm (e.g., ≥160×160 µm). Although the use of a 320×320 µm sampling area (it is larger than 1 degree) represents an extreme option to analyze the photoreceptor mosaic, it is clear from the present study that the window size should be clarified when comparing data between clinical studies.

A moderate agreement was found between density values calculated in sampling areas of the same size at corresponding radial and linear retinal distances. This approach was chosen in order to understand the potential error due to comparing density values calculated at radial or linear distances from the foveal center or PRL in different studies, while assuming an equivalent conversion factor between study populations (e.g., 1 degree = 290 µm). In addition, we found an average distance of 27±15 µm between the PRL and the foveal center. The displacement of the PRL to foveal center was not correlated to SEr and AxL. This result was in fair accordance with previous studies using either AO-flood illuminated or AO-SLO instruments [4], [29], in which the PRL was found to deviate, on average, 18 µm and 34 µm from the foveal center (identified as the peak cone density) respectively. In previous work [10], we showed that by laterally displacing the center of a 50×50 µm sampling window by 18 µm, the displacement error in cone density measurements was 1000 cones/mm^2^ and 500 cones/mm^2^ at 250- and 1300-µm eccentricity, respectively. This type of error was shown to be lower than the accuracy of the cone identification algorithm. Overall, care should be taken when comparing cone density referred to the PRL or foveal center without considering the proportional error related to the window size (the larger the area, the higher the error) and the retinal eccentricity (the closer to the fovea, the higher the error) in different study populations. Caution should be also taken when comparing cone density measured at radial and linear distance from the foveal reference point without taking into consideration the AxL/SEr of the study populations between different studies.

**Figure 4:**
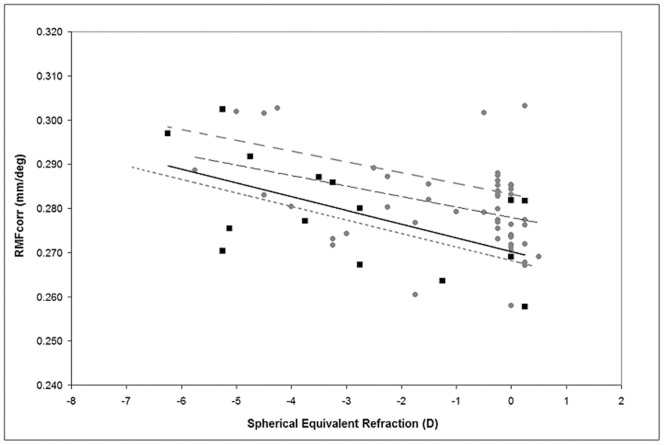
Spectacle corrected Retinal Magnification Factor (RMF_corr_) plotted as a function of the spherical equivalent spectacle correction. The solid line represents linear regression to data from the present work (black squares). The interrupted black line represents linear fit to aggregate data from three of our previous studies (total 62 eyes, gray dots; Lombardo M. et al. Retina, 2013; Lombardo et al. OPO, 2013; Lombardo et al. JCRS, 2013); the interrupted and dotted grey lines represent linear fits to data from Li et al. (18 eyes; IOVS, 2010) and Coletta & Watson (18 eyes; Vis Res, 2006) respectively.

Both a high correlation (R≥0.8) and a high absolute agreement were found between cone density estimated in sampling areas of same size when comparing data calculated with or without using the individual RMF_corr_. This was the only case for which density data, taken within sampling windows of the same size, could be used interchangeably without incurring errors. Overall, cone density has been demonstrated to be fairly constant in eyes with AxL ranging between 22 and 26 mm in previous studies [3], [4], [6], [7], [10], [14]. The difference between the RMF_corr_ values calculated using different model eyes has been shown to be small [3]–[15]. No direct evidence shows which model eye can be considered the most accurate one [17]. Authors have already used a single RMF_corr_, ranging from 0.275 to 0.290 mm/deg, to compensate for the retinal distance from 0.6 to 12 degrees eccentricity [3], [14], [20], [24], [30]. Drasdo and Fowler showed that the RMF value changes less than 0.02 mm/deg from 0 to 10 degrees at the retina of a schematic eye [24]. In this study, the trend observed for the RMF_corr_ in relation to both AxL and SEr was in agreement with previous reports (figure 4) [3], [4], [6], [7], [10], [14], [25]. In an effort to translate AO ophthalmoscopy to a clinically valuable tool, it would be desirable to simplify its use, for example avoiding the need to acquire biometry data in each subject/eye and using a single conversion RMF value. According to previous data [1], [3], [4], [6], [7], [10], [14], [17], [20], [24], [25] and those from the present work, we can propose to use the following RMF_corr_ values for each SEr range when estimating cone parameters in the parafoveal region from 1 to 10 degrees: 0.278 mm/deg in emmetropes/low myopes (SEr range: +0.25 to −1.25 D), 0.282 mm/deg in low/moderate myopes (SEr range: −1.50 to −4.00 D) and 0.292 mm/deg in moderate/high myopes (SEr range: −4.25 to −6.50 D). These values represent the median RMF_corr_ values from previous ex-vivo and in-vivo studies using both AO-flood illuminated and AO-SLO retinal imaging devices.

The analysis of Voronoi diagrams was shown to provide consistent values between sampling areas of different size; the greatest average difference between 6*n* tiles, although not statistically significant, was 2.0% and was found between the 320×320 µm and 64×64 µm areas at 1.5 degree temporal eccentricity. The average % of non hexagonal arrangements was quite similar between sampling windows of different size (difference ≤2.4%), except for the 5*n* tiles across the 320×320 µm and 64×64 µm windows at 418 µm eccentricity (average difference of 3.1%; P<0.05). In addition, the % of 6*n* and 7*n* tiles was almost stable between sampling areas of different size, while the % of 5*n* and 8*n* tended to increase with decreasing window size. It was also valuable to understand that the percentage of 6*n* Voronoi tiles was higher along the superior (>4%) than temporal retinal location.

**Figure 5:**
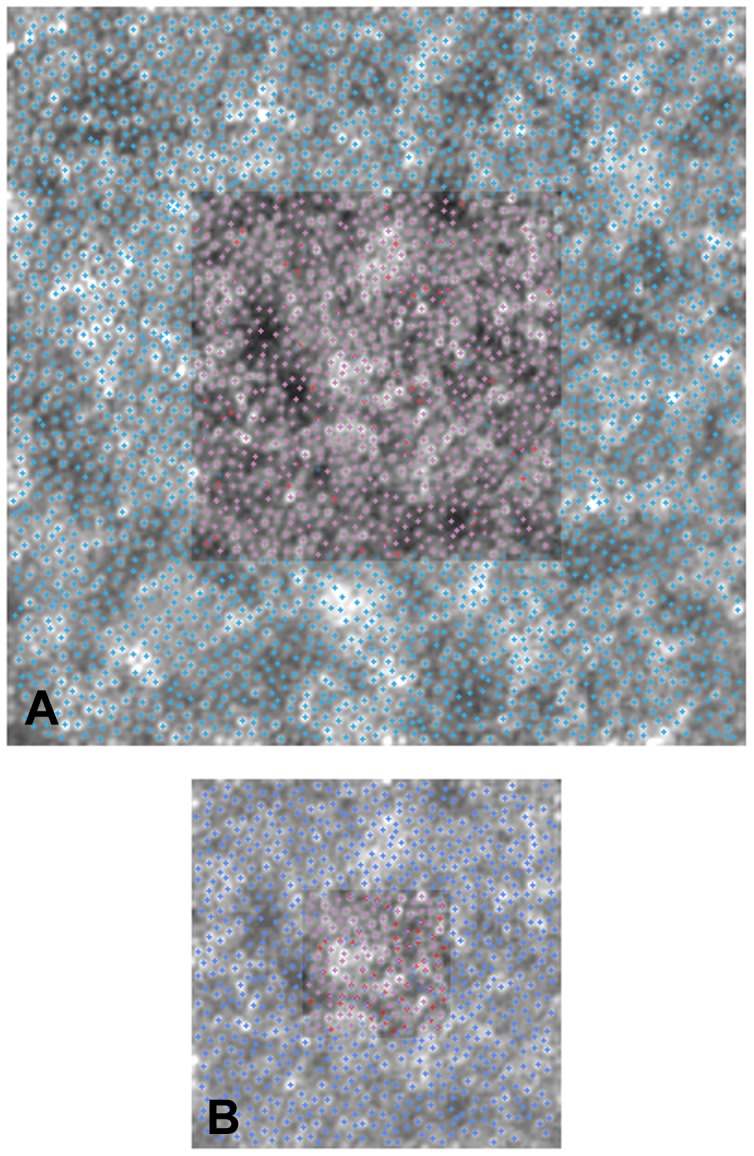
Effect of manual checking of cone identification on Voronoi diagrams. AO retinal images from case W10_S14 (from fig. 2). We revealed the effect of manual re-selection of cones missed by automated identification on variation of the relative arrangements of Voronoi tiles in the same mosaic. Panel A: the central inset shows a 160×160 µm overlapped to a 320×320 µm area. The violet crosses represent the cones that have been identified at exactly the same position in both cases (93%); the red and blue crosses show the cones that have been identified only in 160×160 µm and 320×320 µm area respectively. Panel B: the central inset shows a 64×64 µm overlapped to a 160×160 µm area. 72% cones have been identified at exactly the same position.

In high-quality AO images of the cone mosaic, abnormalities of the cone mosaic have been shown to occur even when estimates of cone density were within normal limits [21], [22], [31]. The results from the present work illustrated the reliability of using Voronoi analysis to compare data of 6*n* preferred packing arrangements taken from sampling areas of different size in the same subject or between subjects [15], [32]. Overall, the graphical representation of the cone mosaic geometry is less sensitive to window size than cone density, as previously shown [15]. This is in principle due to the fact that Voronoi tessellation is not dependent on the window size but only on the number of the labelled cones and their relative arrangements. The difference in percentage of the preferred packing arrangements of cones in sampling areas of different size was in part caused by the re-selection of cones in each sampling window. The number of neighbours between some Voronoi tiles of the same cone mosaic changed because of small differences in the position of cones between each image section, as shown in figure 5. Manual re-selection of the unidentified or misidentified cones was revealed to be one of the sources of variation of preferred cone packing arrangements in Voronoi diagrams. The results cannot be explained by imaging artifacts or bias, because we used high-quality images of the same cone mosaic in each subject/eye. However, the number of manually added cones increased with decreasing window size, likely due to relatively increased proportion of dark areas in the image of the cone mosaic (e.g., retinal vessels etc.) [12], [13], [15]. In order to avoid displacement of point coordinates of the same mosaic and thereby develop reliable Voronoi maps, the cone identification algorithm should segment cone apertures, as recently shown by Chiu et al. [33], and not only their peak intensity. The *undersampling* and the *boundary* effects were found to be additional factors that influence Voronoi analysis of the cone mosaic. Presence of dark areas across the image (e.g., any retinal vessels, rods, non-waveguiding cones etc.), leading to ambiguous identification of cones, has been previously shown to decrease the accuracy of Voronoi analysis [15], [18], [26]. The *boundary* effect increases as sampling area decreases. This phenomenon implies that a higher proportion of cones are close to the boundary with decreasing window size, so that their nearest neighbours in the effective sampling area may not be their real neighbours in the original population [26]. The use of a large buffer zone, wider than used in the present work, can remove this type of error [15], [18], [26].

Cone spacing has not been included in the present analysis, because it has been previously shown to be less sensitive to errors in cone labelling than cone density [12], [13], [34]. Limitations from the present work included the analysis of data obtained from only two retinal locations, although along different retinal meridians. Data obtained at 1.5 degree from the fovea cannot be directly extended to areas closer to the fovea, where cone density is changing most rapidly and therefore differences in cone density estimates between sampling windows of different size could be greater. In addition, the results cannot be generalized to other non commercial AO-flood illuminated systems or to AO-SLO instruments, as discussed previously [13].

In conclusion, we showed the effect of various methodological factors on cone density and packing arrangement estimates in AO flood-illuminated images of the parafoveal cone mosaic. Cone density estimated within sampling areas of different size cannot be compared in clinical studies. The graphical representation of preferred packing arrangements of cones by Voronoi tiles was slightly affected by window size. Assuming that clear and stable identification of each cone in the image is achieved, Voronoi data obtained from sampling areas of different size (using large buffer zone) at corresponding retinal eccentricity can be compared between different subjects and even in the same subject at different time intervals of observation.

## References

[1] Li KY, Roorda A (2007) Automated identification of cone photoreceptors in adaptive optics retinal images. J Opt Soc Am A 24: 1358–1363. 10.1364/josaa.24.001358 17429481

[2] Xue B, Choi SS, Doble N, Werner JS (2007) Photoreceptor counting and montaging of en-face retinal images from an adaptive optics fundus camera. J Opt Soc Am A 24: 1364–1372. 10.1364/josaa.24.001364 PMC2583217 17429482

[3] Song H, Chui TYP, Zhong Z, Elsner AE, Burns SA (2011) Variation of cone photoreceptor packing density with retinal eccentricity and age. Invest Ophthalmol Vis Sci 52: 7376–7384. 21724911 10.1167/iovs.11-7199 PMC3183974

[4] Li KY, Tiruveedhula P, Roorda A (2010) Intersubject variability of foveal cone photoreceptor density in relation to eye length. Invest Ophthalmol Vis Sci 51: 6858–6867. 20688730 10.1167/iovs.10-5499 PMC3055782

[5] Chui TYP, Song H, Burns S (2008) Adaptive-optics imaging of human cone photoreceptor distribution. J Opt Soc Am A 25: 3021–3029. 10.1364/josaa.25.003021 PMC2738986 19037393

[6] Chui TYP, Song H, Burns S (2008) Individual variations in human cone photoreceptor packing density: variations with refractive error. Invest Ophthalmol Vis Sci 49: 4679–4687. 18552378 10.1167/iovs.08-2135 PMC2710765

[7] Lombardo M, Serrao S, Ducoli P, Lombardo G (2012) Variations in the image optical quality of the eye and the sampling limit of resolution of the cone mosaic with axial length in young adults. J Cataract Refract Surg 38: 1147–1155. 22727285 10.1016/j.jcrs.2012.02.033

[8] Merino D, Duncan JL, Tiruveedhula P, Roorda A (2011) Observation of cone and rod photoreceptors in normal subjects and patients using a new generation adaptive optics scanning laser ophthalmoscope. Biomed Opt Express 2: 2189–2201. 21833357 10.1364/BOE.2.002189 PMC3149518

[9] Dees EW, Dubra A, Baraas RC (2011) Variability in parafoveal cone mosaic in normal trichromatic individuals. Biomed Opt Express 2: 1351–1358. 21559146 10.1364/BOE.2.001351 PMC3087591

[10] Lombardo M, Lombardo G, Schiano Lomoriello D, Ducoli P, Stirpe M, et al (2013) Interocular symmetry of parafoveal photoreceptor cone density distribution. Retina 33(8): 1640–1649. 23538574 10.1097/IAE.0b013e3182807642

[11] Lombardo M, Serrao S, Ducoli P, Lombardo G (2012) Adaptive optics photoreceptor imaging, Ophthalmology. 119: 1498–198e2. 10.1016/j.ophtha.2012.03.019 22749092

[12] Garrioch R, Langlo C, Dubis AM, Cooper RF, Dubra A, et al (2012) Repeatability on in vivo cone density and spacing measurements. Optom Vis Sci 89: 632–643. 22504330 10.1097/OPX.0b013e3182540562 PMC3348369

[13] Lombardo M, Serrao S, Ducoli P, Lombardo G (2013) Eccentricity dependent changes of density, spacing and packing arrangement of parafoveal cones. Ophthalmic Physiol Optics 33(4): 516–526. 10.1111/opo.12053 23550537

[14] Park S P, Chung JK, Greenstein V, Tsang SH, Chang S (2013) A study of factors affecting the human cone photoreceptor density measured by adaptive optics scanning laser ophthalmoscope. Exp Eye Res 108: 1–9. 23276813 10.1016/j.exer.2012.12.011 PMC4388135

[15] Lombardo M, Serrao S, Ducoli P, Lombardo G (2013) Influence of sampling window size and orientation on parafoveal cone packing density. Biomed Opt Express 4(8): 1318–1331. 24009995 10.1364/BOE.4.001318 PMC3756574

[16] Talcott KE, Ratman K, Sundquist SM, Lucero AS, Lujan BJ, et al (2011) Longitudinal study of cone photoreceptors during retinal degeneration and in response to ciliary neurotrophic factor treatment. Invest Ophthalmol Vis Sci 52: 2219–2226. 21087953 10.1167/iovs.10-6479 PMC3080173

[17] Lombardo M, Serrao S, Devaney N, Parravano M, Lombardo G (2013) Adaptive optics technology for high-resolution retinal imaging. Sensors 13: 334–366. 10.3390/s130100334 PMC3574679 23271600

[18] Lombardo M, Parravano M, Serrao S, Boccassini B, Varano M, et al (2014) Adaptive optics imaging of parafoveal cones in type 1 diabetes. Retina 34(3): 546–557. 23928676 10.1097/IAE.0b013e3182a10850

[19] Curcio CA, Sloan KR (1992) Packing geometry of human cone photoreceptors: variation with eccentricity and evidence of local anisotropy. Visual Neurosci 9: 169–180. 10.1017/s0952523800009639 1504026

[20] Curcio CA, Sloan KR, Kalina RE, Hendrickson AE (2009) Human photoreceptor topography. J Comp Neurol 292: 497–523. 10.1002/cne.902920402 2324310

[21] Godara P, Wagner-Schuman M, Rha J, Connor TB Jr, Stepien KE, et al (2012) Imaging the photoreceptor mosaic with adaptive optics: beyond counting cones. Adv Exp Med Biol 723: 451–458. 22183364 10.1007/978-1-4614-0631-0_57 PMC3325514

[22] Boretsky A, Khan F, Burnett G, Hammer DX, Ferguson RD, et al (2012) In vivo imaging of photoreceptor disruption associated with age-related macular degeneration: a pilot study. Laser Surg Med 44: 603–610. 10.1002/lsm.22070 PMC3593748 22930575

[23] Kulcsar C, Le Besnerais G, Odlund E, Levecq X (2013) Robust processing of images sequences produced by an adaptive optics retinal camera. In Imaging and Applied Optics, OSA Technical Digest (available online), paper OW3A.

[24] Drasdo N, Fowler CW (1974) Non-linear projection of the retinal image in a wide-angle schematic eye. Br J Ophthalmol 58: 709–714. 4433482 10.1136/bjo.58.8.709 PMC1215006

[25] Coletta NJ, Watson T (2006) Effect of myopia on visual acuity measured with laser interference fringes. Vis Res 46: 636–651. 16045959 10.1016/j.visres.2005.05.025

[26] Rodieck RW (1991) The density recovery profile: a method for the analysis of points in the plane applicable to retinal studies. Visual Neurosci 6: 95–111. 10.1017/s095252380001049x 2049333

[27] Bland JM, Altmann DG (1986) Statistical methods for assessing agreement between two methods of clinical measurement. Lancet 1: 307–310. 2868172

[28] Bland JM, Altmann DG (1999) Measuring agreement in method comparison studies. Stat Methods Med Res 8: 135–160. 10501650 10.1177/096228029900800204

[29] Putnam NM, Hofer HJ, Doble N, Chen L, Carroll J, et al (2005) The locus of fixation and the foveal cone mosaic. J Vision 5: 632–639. 10.1167/5.7.3 16231998

[30] Holden AL, Fitzke FW (1988) Image size in the fundus: structural evidence for wide-field retinal magnification factor. Br J Ophthalmol 72: 228–230. 3355810 10.1136/bjo.72.3.228 PMC1041414

[31] Da Fontoura Costa L, Oliveira Bonci DM, Saito CA, De Farias Rocha FA, De Lima Silveira LC, et al (2005) Voronoi analysis uncovers relationship between mosaics of normally placed and displaced amacrine cells in the thraira retina. Neuroinformatics 5: 59–77. 10.1385/ni:5:1:59 17426353

[32] Zayit-Soudry S, Duncan JL, Syed R, Menghini M, Roorda AJ (2013) Cone structure with adaptive optics scanning laser ophthalmoscopy in eyes with non-neovascular age-related macular degeneration. Invest Ophthalmol Vis Sci 54(12): 7498–7509. 24135755 10.1167/iovs.13-12433 PMC3832216

[33] Chiu SJ, Lokhnygina Y, Dubis AM, Dubra A, Carroll J, et al (2013) Automatic cone photoreceptor segmentation using graph theory and dynamic programming. Biomed Opt Express 4(6): 924–937. 23761854 10.1364/BOE.4.000924 PMC3675871

[34] Duncan JL, Zhang Y, Gandhi J, Nakanishi C, Othman M, et al (2007) High-resolution imaging with adaptive optics in patients with inherited retinal degeneration. Invest Ophthalmol Vis Sci 48: 3283–329. 17591900 10.1167/iovs.06-1422

